# Crystal structures of the human IgD Fab reveal insights into C_H_1 domain diversity

**DOI:** 10.1016/j.molimm.2023.05.006

**Published:** 2023-07

**Authors:** Anna M. Davies, Rebecca L. Beavil, Momchil Barbolov, Balraj S. Sandhar, Hannah J. Gould, Andrew J. Beavil, Brian J. Sutton, James M. McDonnell

**Affiliations:** a King’s College London, Randall Centre for Cell and Molecular Biophysics, New Hunt’s House, London, SE1 1UL, United Kingdom; b Current address: 272BIO Limited, The Pirbright Institute, B-Block, Ash Road, Pirbright, Woking, Surrey, GU24 0NF, United Kingdom; c Current address: Medical University of Varna, Faculty of Pharmacy, Department of Biochemistry, bul. Tsar Osvoboditel 150, Varna, 9002, Bulgaria; d William Harvey Research Institute, Barts and The London School of Medicine and Dentistry, Queen Mary University of London, London, EC1M 6BQ, United Kingdom

**Keywords:** IgD, CH1 domain, Fab, Antibody, immunoglobulin, X-ray crystallography

## Abstract

Antibodies of the IgD isotype remain the least well characterised of the mammalian immunoglobulin isotypes. Here we report three-dimensional structures for the Fab region of IgD, based on four different crystal structures, at resolutions of 1.45-2.75 Å. These IgD Fab crystals provide the first high-resolution views of the unique Cδ1 domain. Structural comparisons identify regions of conformational diversity within the Cδ1 domain, as well as among the homologous domains of Cα1, Cγ1 and Cµ1. The IgD Fab structure also possesses a unique conformation of the upper hinge region, which may contribute to the overall disposition of the very long linker sequence between the Fab and Fc regions found in human IgD. Structural similarities observed between IgD and IgG, and differences with IgA and IgM, are consistent with predicted evolutionary relationships for the mammalian antibody isotypes.

## 1 Introduction

Described as an enigmatic antibody isotype, and previously thought to have evolved only relatively recently, immunoglobulin D (IgD) is now appreciated to be as ancient as IgM (Ohta, Flajnik, 2006; Chen, Cerutti, 2010). However, in contrast to IgM, the Fab, hinge and Fc regions of IgD display such remarkable structural diversity between species that it has been dubbed “the adaptive immune system’s plaything” (Ohta, Flajnick, 2006). For example, each heavy chain in murine IgD-Fc comprises only a single constant domain, in human IgD-Fc there are two constant domains, while in the Fc regions of IgD from bony fish there are up to 13 constant domains. Human IgD has a 64 residue hinge between the Fabs and Fc region whereas IgD from cold blooded vertebrates lacks a hinge; a longer Fc region is instead suggested to provide flexibility. Furthermore, in many bony fish and artiodactyl species, the Cδ1 domain is even replaced by a Cμ1 or Cμ1-like domain (Ohta, Flajnik, 2006; Chen, Cerutti, 2011; Edholm et al, 2011).

Compared with other isotypes, the role of IgD in humans is less well understood. Membrane-bound IgD, as part of the B-cell receptor for antigen, appears to play a role in B-cell anergy in the periphery (Duty et al. 2009; Gutzeit et al., 2018). By contrast, a significant population of B-cells in the upper respiratory tract expresses membrane-bound IgD, where secreted IgD is suggested to play a role in mucosal immunity. Secreted IgD not only recognizes commensal bacteria that reside in the upper respiratory tract, but can bind to and activate basophils (Chen 2009; Edholm et al, 2011; Gutzeit et al., 2018). Moreover, commensal bacteria such as *Moraxella catarrhalis* have also been shown to activate B-cells in a superantigen-like manner through binding and cross-linking the IgD B-cell receptor by the *Moraxella* IgD-binding protein MID (Jendholm et al., 2009).

IgD is also the antibody isotype that is least well-characterized structurally. X-ray scattering studies have revealed that human IgD is predominantly T-shaped in solution, with a semi-extended hinge (Sun et al., 2005). However, in contrast to other isotypes, there is no high resolution structural information available for any region of IgD.

Here we report four crystal structures of the human IgD Fab that provide the first high resolution views of IgD. Consistent with Fab structures for other isotypes, these structures reveal a range of elbow angles between the V_H_-V_L_ and Cδ1-Cλ domain pairs, in addition to conformational diversity in particular Cδ1 domain loop regions. The Fab structures also reveal that the IgD upper hinge adopts a unique conformation among the human isotypes, which may play a role in determining its overall structure.

## 2 Methods and Materials

### 2.1 Protein expression

The vector encoding the recombinant human IgD Fab was based on a pVITRO plasmid that contained inserts encoding the heavy and light chains of an anti-*Phl p* 7 (grass pollen allergen) IgD antibody. This plasmid had been made according to a previously described method (Dodev et al., 2014) using a pVITRO plasmid encoding a human anti-*Phl p* 7 IgE antibody as a template and an IgD insert synthesized commercially. To generate the construct for the IgD Fab, the IgD heavy chain gene was mutated to incorporate a stop codon after the C-terminus of the Cδ1 domain using a QuikChange Lightning Site-Directed Mutagenesis kit (Agilent) according to the manufacturer’s instructions. Recombinant human IgD Fab was expressed in stably transfected HEK293F cells cultured in spinner flasks in high glucose DMEM supplemented with 10% FCS, 120 U/mL penicillin, 100µg/mL streptomycin, 50µg/mL hygromycin and 2mM L-glutamine. The supernatant was harvested by centrifugation at 4000 x g, followed by filtration using a 0.45μm filter and the addition of 0.1% (w/v) sodium azide.

### 2.2 Protein purification

The IgD Fab was purified by affinity chromatography using the recombinant *Phl p* 7 antigen, which was produced as described previously (Mitropoulou et al., 2018) and then coupled to NHS-activated sepharose (Cytiva), according to the manufacturer’s instructions. After the supernatant was loaded, the column was washed with PBS containing 0.1% (w/v) sodium azide and the Fab was eluted using 0.1M glycine pH 2.5. Fractions were pooled, concentrated and further purified by size-exclusion chromatography using a Superdex 200 Increase column that had been equilibrated with TBS and 0.1% (w/v) sodium azide.

### 2.3 Crystallization

For crystallization trials, the IgD Fab was buffer-exchanged into a buffer containing 100mM Tris pH 7.5 and 50mM NaCl and concentrated to 4.1mg/mL. All crystals were grown at 18°C in SWISSCI MRC 96 well plates using a reservoir volume of 100µL and drops comprising 100nL protein and 100nL reservoir solution. Fab^1^ crystals were grown in 20% (w/v) PEG 3350 and 0.2M sodium phosphate, and were cryoprotected with 28% (w/v) PEG 3350 and 17% (v/v) ethylene glycol before flash-cooling in liquid nitrogen. Fab^2^ crystals were grown in 0.1M BTP pH 6.5, 20% (w/v) PEG 3350 and 0.2M sodium nitrate, and were cryoprotected with 28% (w/v) PEG 3350 and 17% (v/v) ethylene glycol. Fab^3^ crystals were grown in 16% (w/v) PEG 6000 and 0.1M tri-sodium citrate, and were cryoprotected with 25% (w/v) PEG 6000, 0.1M tri-sodium citrate and 17% (v/v) ethylene glycol. Fab^4^ crystals were grown in 0.1M Tris pH 8.5, 18% (w/v) PEG 8000 and 0.2M lithium sulphate, and were cryoprotected with 0.1M Tris pH 8.6, 25% (w/v) PEG 8000 and 15% (v/v) ethylene glycol.

### 2.4 X-ray diffraction data collection, structure determination and refinement

Data were collected at beamline I04-1 at the Diamond Light Source (Harwell, UK). Intensities were integrated using the xia2 3dii or xia2 DIALS automatic processing pipelines at Diamond (Winter, 2010; Winter et al., 2018) and further processed with programs from the CCP4 suite (Winn et al., 2011; Evans and Murshudov, 2013). The Fab^3^ structure was solved first by molecular replacement with PHASER (McCoy et al., 2007), using Fab protein atoms from Protein Data Bank (PDB) entry 5OTJ (Mitropoulou et al., 2018) as a search model. Structures Fab^1^, Fab^2^ and Fab^4^ were then solved by molecular replacement using protein atoms from the Fab^3^ structure as a search model. The structures were refined with PHENIX (Liebschner et al., 2019) and manual model building was performed with Coot (Emsley et al., 2010). The quality of the structures was assessed with MolProbity (Williams et al., 2018). Data processing and refinement statistics are summarized in Table 1. Fab elbow angles were calculated using the RBOW program (Stanfield et al., 2006). Figures were produced with PyMOL (The PyMOL Molecular Graphics System, Version 1.1r1, Schrödinger, LLC).

### 2.5 Structural comparison

C_H_1 domain protein sequences for Cα1 (subclass IgA1), Cγ1 (subclasses IgG1, IgG2, IgG3 and IgG4) and Cμ1 domains were obtained from UniProt (The UniProt Consortium, 2021) and used to search the PDB. Structures of the human IgA2 Cε1 and IgE C∊1 domains have not yet been reported. PDB entries returned from the search were sorted by resolution, from highest to lowest. An initial set of up to 10 structures solved at 2.7Å resolution or higher, and belonging to different space groups, or the same space group but with different unit cell dimensions, was then selected for each isotype. Within each set, structures were then superposed on the C_H_1 domain. Where a structure contained more than one molecule in the asymmetric unit, every molecule was treated as an additional, independent structure. For each isotype, a smaller sub-set was then selected that included a representative set of conformations in the C_H_1 domain loop regions. For structures in which all loop conformations were similar, only one structure (typically the one solved at highest resolution) was included in the representative set for that particular isotype. The following structures were included: IgA1, PDB entries 3M8O, 3QNX and 3QNY (Correa et al., 2013); IgG1, PDB entries 5WCA (Andrews et al., 2017), 6BJZ (Henderson et al., 2020), 6OC7 (Steichen et al., 2019), 6UCF (Shen et al., 2020), 6VI2 (Lokareddy et al., 2020), 6WFY (Pholcharee et al., 2021), 7K3Q (Tortorici et al., 2020) and 7OW1 (Bakrania et al., 2022); IgG2, PDB entries 4EDW (La Porte et al., 2014), 4HCR (Yu et al., 2013), 5J13 (Verstraete et al., 2017), 6TKC and 6TKD (Orr et al., 2022); IgG3, PDB entries 1Q1J (Stanfield et al., 2004), 3C2A (Dhillon et al., 2008), 3GHB (Burke et al., 2009) and 4M1D (Killikelly et al., 2013); IgG4, PDB entries 4DTG (Hilden et al., 2012), 5DK3 (Scapin et al., 2015), 6GFE (Blech et al., 2019), 6K0Y (Liu et al., 2019), 7Q4Q (Gutiérrez-Fernández et al., 2022) and 7VUX (Zhao et al., 2022); IgM, PDB entries 1DEE (Graille et al., 2000), 1DN0 (Cauerhff et al., 2000), 1HEZ (Graille et al., 2001) and 2AGJ (Ramsland et al., 2006). Loop regions were defined according to Halaby et al., 1999. C_H_1 domain residue numbers are based on the numbering system used in the structures of the IgD Fab.

## Results and Discussion

### 3.1 Overall structure of the human IgD Fab

We solved four crystal structures of a human anti-*Phl p* 7 IgD Fab: Fab^1^ (2.75Å resolution) solved in space group *P* 1 with two Fab molecules (Fab^1A^ and Fab^1B^) in the asymmetric unit, Fab^2^ and Fab^3^ (1.55Å and 1.45Å resolution, respectively), solved in space group *P* 2_1_ with one molecule each in the asymmetric unit, and Fab^4^ (2.1Å resolution) solved in space group *P* 2_1_ 2_1_ 2_1_ with one molecule in the asymmetric unit.

**Fig. 1:**
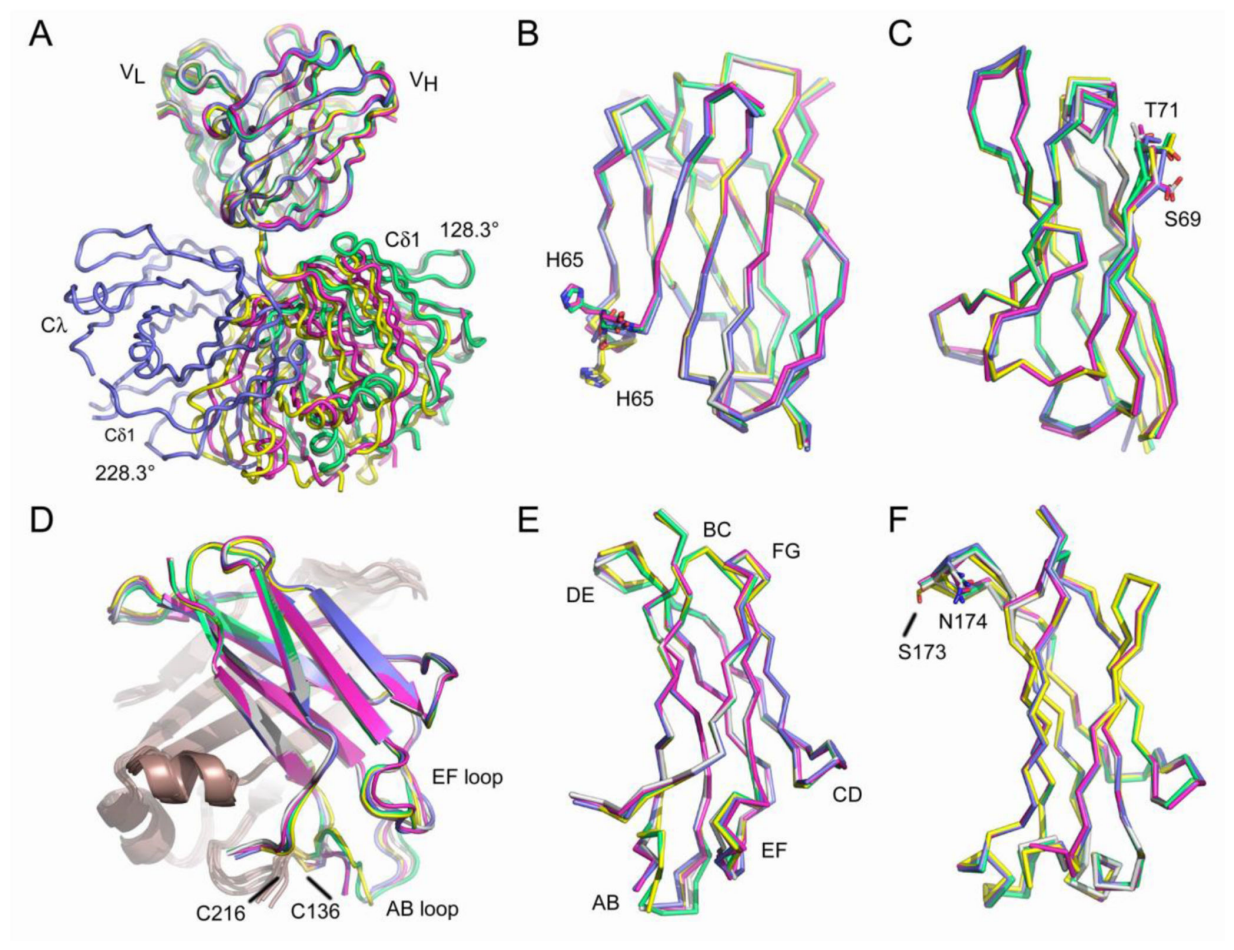
Overall structure of the IgD Fab. (A) A range of elbow angles (128.3-228.3°) were observed between the V_H_-V_L_ and Cδ1-Cλ domain pairs. IgD Fab molecules were superposed on V_H_ domain Cα atoms. (B) Superposition of V_H_ domain Cα atoms revealed a conformational change about Gly66 that altered the position of His65. (C) Superposition of V_L_ domain Cα atoms revealed a conformational change about Gly70 that altered the positions of Ser69 and Thr71. (D) Superposition of Cδ1 domain Cα atoms revealed conformational differences in the AB and EF loops. The cysteine residues involved in forming the disulfide bond between heavy (C136) and light (C216) chains are labelled. The light chain is colored in bronze. (E) View of the Cδ1 domain. The loop regions are labelled. (F) Superposition of Cλ domain Cα atoms revealed a small shift in the DE loop that altered the positions of Ser173 and Asn174. In panels A-F, individual domains and IgD Fab molecules are coloured as follows: Fab^1A^, purple; Fab^1B^, blue; Fab^2^, green; Fab^3^, grey; Fab^4^, yellow.

Like Fab fragments in other antibody isotypes (Stanfield et al., 2006), the crystal structures of the IgD Fab reveal a range of Fab elbow angles between the V_H_-V_L_ and Cδ1-Cλ domain pairs, from 128.3° (Fab^3^) to 228.3° (Fab^1B^) (Fig. 1A). The ABangle tool (Dunbar et al., 2013) was used to measure the set of five angles (HL, HC1, LC1, HC2 and LC2) and one distance (dc) that describe the orientation of the VH and VL domains. The range of values measured for the human IgD Fab were: HC1, 56.6° (Fab^1B^ and Fab^3^) to 57.3° (Fab^2^); HC2, 107.2° (Fab^1B^) to 111.5° (Fab^3^); HL, -62.1° (Fab^3^) to -64.3° (Fab^1A^); LC1, 100.5° (Fab^1A^) to 105.4° (Fab^3^); LC2, 78.3° (Fab^1B^) to 82.3° (Fab^4^); dc, 18.3Å (Fab^2^) to 19.2Å (Fab^1B^).

Using the highest resolution structure (Fab^3^) as the reference, Cα atoms from the V_H_ domain of each structure were superposed with an RMSD range of 0.41-0.63Å (Fig. 1B). The conformations of CDRH1-3 were similar, and the most significant difference between the structures was a conformational change about Gly66 in the C”-D loop, which altered the position of His65. Cα atoms from the V_L_ domain were superposed with an RMSD range of 0.35-0.50Å (Fig. 1C). The most significant difference between the structures was a conformational change about Gly70 in the DE loop, which altered the positions of Ser69 and Thr71. Cα atoms from the Cδ1 domain were superposed with an RMSD range of 0.48-0.78Å (Figs. 1D and 1E), revealing modest conformational differences in the AB and EF loops, which are discussed in further detail below. The Cδ1 domain BC, CD, DE and FG loops adopted similar conformations in all five molecules. Cα atoms from the Cλ domain were superposed with an RMSD range of 0.21-0.63Å (Fig. 1F). The structures revealed some variation in the DE loop, which altered the positions of the Ser173 and Asn174 Cα atoms by ~1.8Å. Although some of the loop regions in some of the Fab structures form crystal packing interactions, the different conformations could not be attributed to differences in crystal contacts.

### 3.2 Structural diversity in the Cδ1 domain

**Fig. 2:**
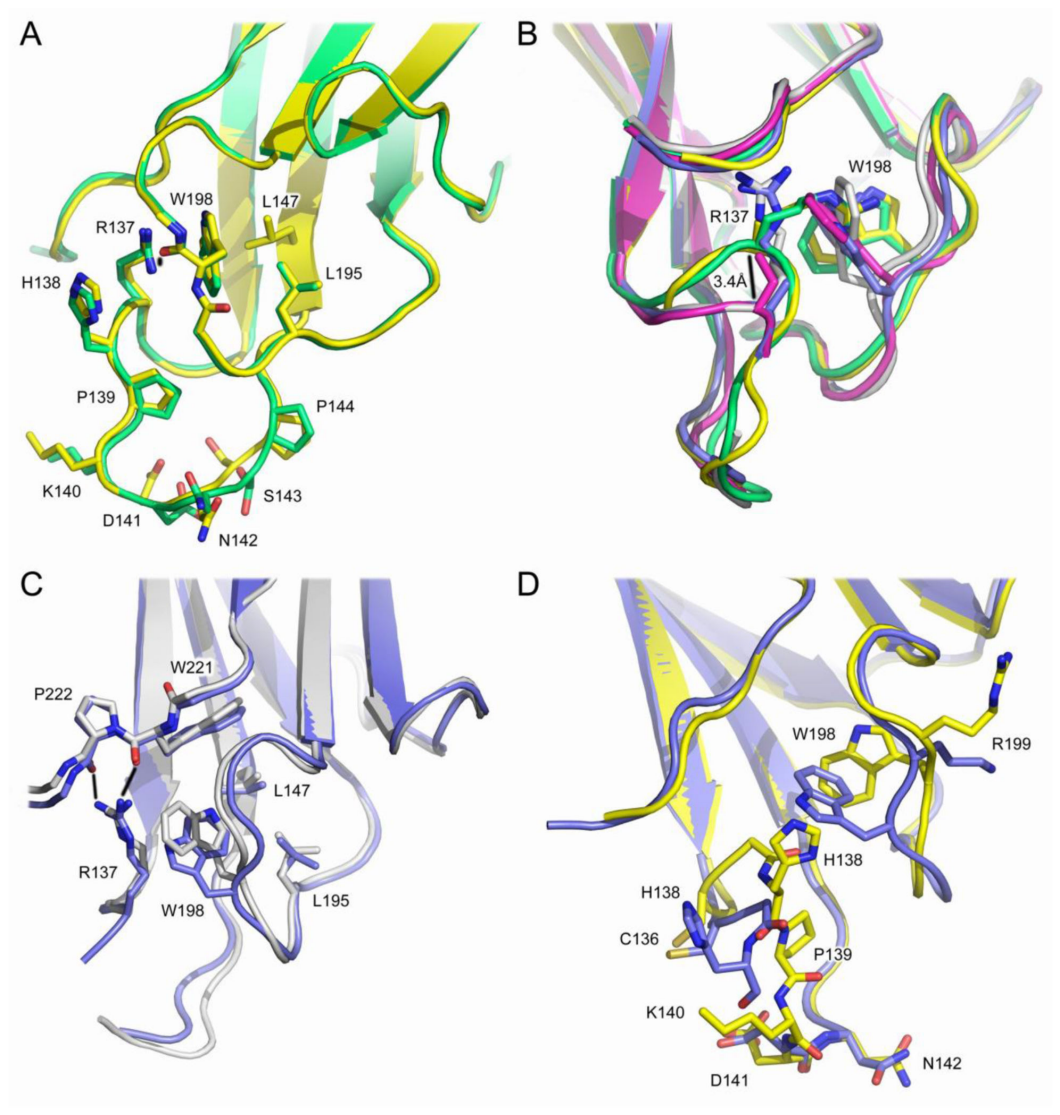
Structural diversity in the Cδ1 domain. (A) Cδ1 domain AB loop structure in Fab^2^ (green) and Fab^4^ (yellow). The plane of the Arg137 guanidinium group is parallel to the plane of the Trp198 indole group. Arg137 forms a hydrogen bond (depicted by a black line) with the Trp198 main chain. (B) In Fab^1B^ (blue) and Fab^3^ (grey), the plane of the Trp198 indole group is almost perpendicular to its position in Fab^2^ (green) and Fab^4^ (yellow). In Fab^1A^ (purple), Fab^1B^ and Fab^3^, the position of the R137 Cα atom is shifted by ~3.4Å (depicted by a black line) compared with its position in Fab^2^ and Fab^4^. (C) In Fab^1B^ (blue) and Fab^3^ (grey), the Arg137 side chain forms hydrogen bonds (depicted by black lines) with Trp221 and Pro222 main chain atoms. The overall orientation of the Trp198 indole group differs in these structures. (D) The conformations of other AB loop residues, such as Cys136 and His138, also differ between the different structures, such as those for Fab^1B^ (blue) and Fab^4^ (yellow) shown here. In Fab^1B^, AB loop residues 139 and 140 are disordered. In panels A-D, the IgD Fab molecules are coloured as follows: Fab^1A^, purple; Fab^1B^, blue; Fab^2^, green; Fab^3^, grey; Fab^4^, yellow. For clarity, the light chain has not been shown.

Structural diversity in the Cδ1 domain is seen in the AB (residues 133-146) and EF (residues 193-202) loop regions, particularly around Arg137 (AB loop) and Trp198 (EF loop). In Fab^2^ and Fab^4^, Trp198 packs against Leu147 and Leu195, the plane of the Arg137 guanidinium group is parallel to the plane of the Trp198 indole group, and the Arg137 side chain forms a hydrogen bond with the Trp198 main chain (Fig. 2A). By contrast, in Fab^1B^ and Fab^3^, the plane of the Trp198 indole group is almost perpendicular to its position in Fab^2^ and Fab^4^; in Fab^1B^ and Fab^3^ the Trp198 side chain occupies a similar space, but the overall orientation of the indole group differs. (Figs. 2B and 2C). The position of Trp198 in Fab^1B^ and Fab^3^ would clash with the position of Arg137 observed in Fab^2^ and Fab^4^ (Fig. 2B). Instead, in Fab^1B^ and Fab^3^, the Arg137 side chain faces Pro222, and forms hydrogen bonds with Trp221 and Pro222 main chain atoms (Fig. 2C). In these molecules, Arg137 still contacts the Trp198 side chain, but the position of the Arg137 Cα atom is shifted by ~3.4Å compared with Fab^2^ and Fab^4^ (Fig. 2B). The plane of the Trp198 indole group is rotated in Fab^1A^ compared with its position in Fab^1B^ and Fab^3^, and Arg137 is partially disordered, but the Arg137 Cα atom is shifted as in Fab^1B^ and Fab^3^ (Fig. 2B).

In Fab^1A^, Fab^1B^ and Fab^3^, other AB loop residues also undergo conformational changes compared with Fab^2^ and Fab^4^. For example, even though the disulfide bond with Cys216 from the λ light chain is unaffected, the position of the Cys136 Cα atom differs by up to 1.9A. His138 is disordered in Fab^3^ but undergoes a substantial conformational change in Fab^1A^ and Fab^1B^, with the position of its Cα atom shifted by ~3.9Å compared with that in Fab^2^ and Fab^4^. Part of the AB loop is also disordered in Fab^1A^, Fab^1B^ and Fab^3^ (Fig. 2D); although the AB loop is ordered in Fab^2^ and Fab^4^, the structure of the loop differs between these two Fab molecules (Fig. 2A).

In the EF loop, the most significant difference between the five Fab molecules, apart from the conformational change involving Trp198, is the position adopted by Arg199 (Fig. 2D). In Fab^2^, Fab^3^ and Fab^4^, the Arg199 side chain extends away from the Cδ1 domain. By contrast, in Fab^1A^ and Fab^1B^, the Arg199 side chain is oriented towards the surface of the Cδ1 domain.

Residues 198-206, which form part of the Cδ1 domain EF loop and β-strand F, are reported to form part of the binding site for the *Moraxella catarrhalis* IgD-binding protein MID (Samuelsson et al., 2006). However, it remains to be determined whether this structural diversity in the Cδ1 domain, and the MID binding site, has any functional implications.

### 3.3 Conformational diversity in human C_H_1 domain loop regions

**Fig. 3:**
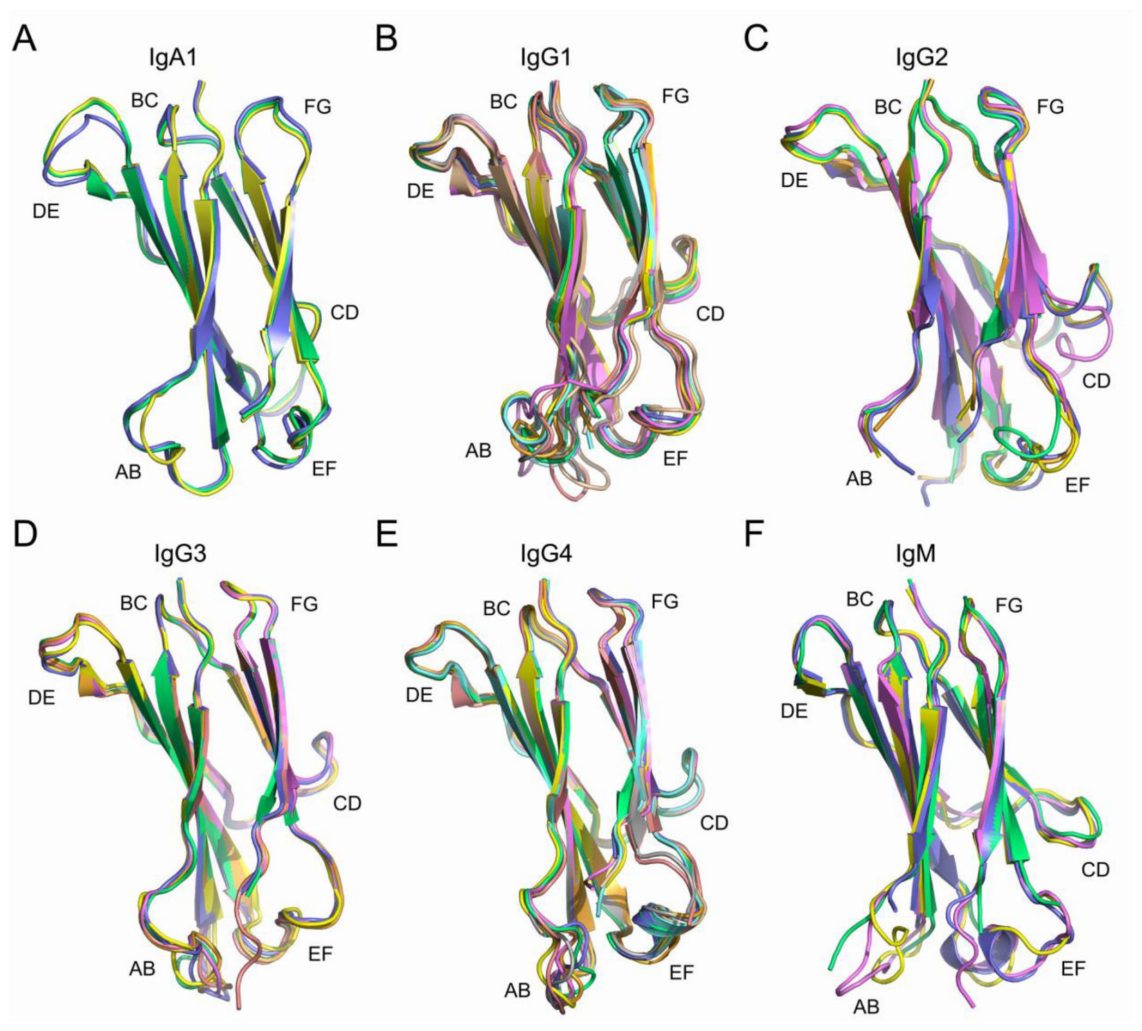
Conformational diversity in human C_H_1 domain loop regions. (A) IgA1. The Cα1 domain AB and DE loops are conformationally diverse. Structures are coloured as follows: PDB entry 3M8O, blue; 3QNX, yellow; 3QNY, green. (B) IgG1. The Cγ1 domain AB loop is conformationally diverse, while the EF loop exhibits backbone shifts. Structures are coloured as follows: PDB entry 5WCA, yellow; 6BJZ, green; 6OC7, purple; 6UCF, orange; 6VI2^Fab1^, salmon; 6VI2^Fab2^, grey; 6WFY, blue; 7K3Q, aquamarine; 7OW1, wheat. (C) IgG2. The Cγ1 domain CD and EF loops are conformationally diverse. Structures are coloured as follows: PDB entry 4EDW, yellow; 4HCR, green; 5J13, purple; 7TKC, orange; 6TKD, blue. (D) IgG3. The Cγ1 domain AB loop is conformationally diverse, while the DE and EF loops exhibit backbone shifts. Structures are coloured as follows: PDB entry 1Q1J, green; 3C2A^Fab1^, purple; 3C2A^Fab2^, orange; 3GHB, salmon; 4M1D^Fab1^, blue; 4M1D^Fab2^, yellow. (E) IgG4. The Cγ1 domain AB and EF loops are conformationally diverse. Structures are coloured as follows: PDB entry 4DTG, yellow; 5DK3^Fab1^, salmon; 5DK3^Fab2^, grey; 6GFE^Fab1^, aquamarine; 6GFE ^Fab2^, wheat; 6K0Y, green; 7Q4Q^Fab1^, purple; 7Q4Q^Fab2^, orange; 7VUX, blue. (F) IgM. The Cμ1 domain AB and EF loops are conformationally diverse. Structures are coloured as follows: PDB entry 1DEE, yellow; 1DN0, blue; 1HEZ, green; 2AGJ, purple. Where a structure contained more than one Fab molecule in the asymmetric unit, and both were included in the analysis, the molecules were designated ^Fab1^ and ^Fab2^.

A set of crystal structures solved for other human isotypes (IgA1, IgG1-4 and IgM) was selected and analysed to assess their structural diversity. Like the IgD Cδ1 domain (Figs. 1D and 1E), the core of the immunoglobulin fold is conserved in Cα1 (subclass IgA1), Cγ1 (subclasses IgG1-IgG4) and Cμ1 domains. Conformational diversity in the C_H_1 domain loops was observed in each isotype and subclass as follows: in the AB and DE loops in IgA1 (Fig. 3A); in the AB loop, together with backbone variations in the EF loop, in IgG1 (Fig. 3B); in the CD and EF loops in IgG2 (the AB loop was partially disordered in the IgG2 structures) (Fig. 3C); in the AB loop, together with backbone variations in the DE and EF loops, in IgG3 (Fig. 3D); in the AB and, to a lesser extent, EF loops in IgG4 (Fig. 3E) and the AB and EF loops in IgM (Fig. 3F). With the exception of the DE loop in IgA1, the overall conformations of the BC, DE and FG loops were relatively conserved within each isotype and subclass (Figs. 3A-F).

### 3.4 The Cδ1 domain BC, DE and FG loop conformations show structural similarities between IgD and IgG and differences with IgA and IgM

The precise evolutionary relationships between the five human antibody isotypes are still not fully understood. However, IgD is suggested to have given rise to the IgY isotype (in amphibians, birds and reptiles), from which mammalian IgG and IgE are thought to have evolved (Estevez et al., 2016; Hordvik et al., 1999; Zhang et al., 2017). IgM is the ancestor of IgA, which evolved along a different path (Zhang et al., 2017). Consistent with these proposed evolutionary relationships, the BC, DE and FG loops regions of IgD are similar to IgG but different from IgA and IgM.

**Fig. 4:**
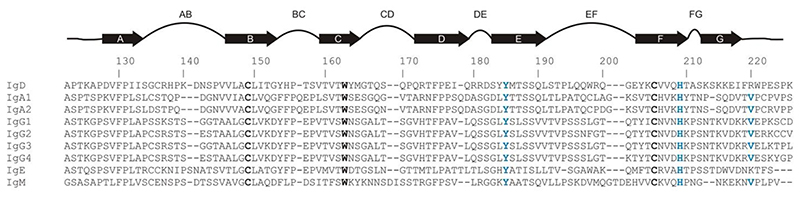
Alignment of human C_H_1 domain sequences. Sequences were obtained from UniProt (The UniProt Consortium, 2021) with the following accession numbers: IgA1, P01876; IgA2, P01877; IgD, P01880; IgE, P01854; IgG1, P01857; IgG2, P01859; IgG3, P01860; IgG4, P01861; IgM, P01871. For all sequences, the numbering system from the IgD Fab structures has been used. The sequence alignment was performed using Clustal Omega (Madeira et al., 2022). Small manual adjustments were made to the sequence alignment after structural comparisons were performed to ensure better agreement between the alignment and structures. Highly conserved amino acids of the ‘central pin’ residues of Ig-domains (Williams and Barclay, 1988) are in bold and coloured black. Other noteworthy, conserved residues in C_H_1 domains are coloured blue. The IgM Cμ1 FG loop has two fewer residues compared with IgD and IgG and a two-residue gap was inserted into the IgM sequence after position 212. In the Clustal Omega sequence alignment, a gap that had been inserted in the IgM Cμ1 β-strand G at position 217 was removed. The IgA Cα1 FG loop has one fewer residue compared with IgD and IgG and a single residue gap was inserted into the IgA sequence after position 213.

**Fig. 5:**
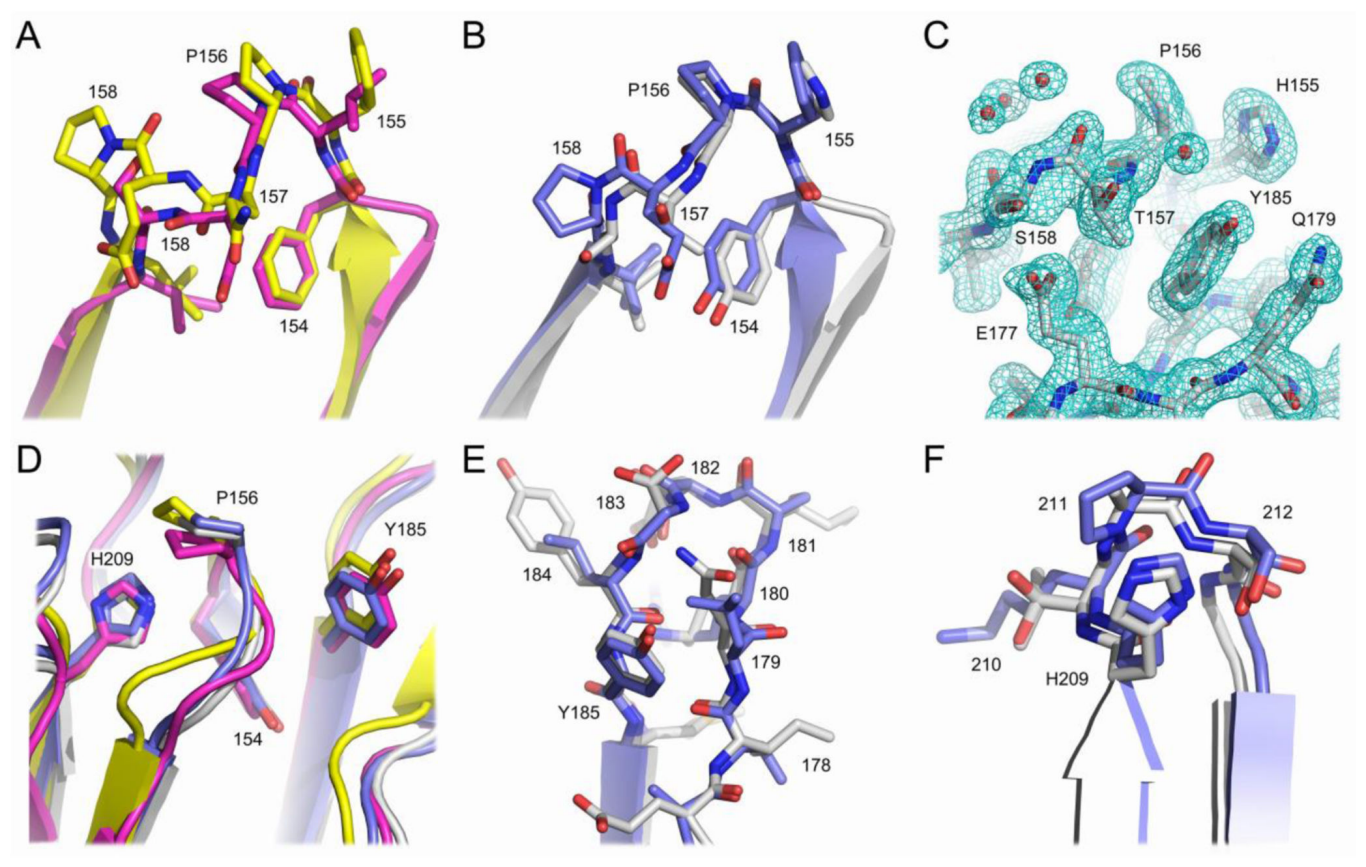
The C_H_1 BC, DE and FG loop structures are similar in IgD and IgG. (A) C_H_1 BC loop structure in IgA1 (PDB entry 3M8O, yellow) and IgM (PDB entry 1DN0, purple). The *cis* peptide bond between residue 155 and Pro156 is shown. In IgA1, a *cis* peptide bond is also found between the glutamic acid insertion and Pro158. As the loop structure is similar within each isotype, a single representative structure has been shown. (B) C_H_1 BC loop structure in IgG1 (PDB entry 6WFY, blue) and IgD (Fab^3^, grey). The cis peptide bond between residue 155 and Pro156 is shown. In both IgG1 and IgD, a *cis* peptide bond is found between residues 157 and 158. As the overall loop structure is similar within each IgG subclass, and between IgD and IgG isotypes, one representative structure has been shown for each of the Cδ1 and Cγ1 domains. (C) Electron density for part of the Cδ1 BC and DE loops, including the *cis* peptide bond between Thr157 and Ser158. A 2F_o_-F_c_ map is shown, contoured at 1 . (D) In IgA1 (PDB entry 3M8O, yellow), IgD (Fab^3^, grey), IgG1 (PDB entry 6WFY, blue) and IgM (PDB entry 1DN0, purple), a conserved tyrosine residue in the DE loop at position 185 and a conserved histidine residue in the FG loop at position 209 pack against the BC loop. As the positions of the conserved residues are similar in each isotype (and within each IgG subclass), a single representative structure has been shown for each isotype. (E) C_H_1 DE loop structure in IgG1 (PDB entry 6WFY, blue) and IgD (Fab^3^, grey). The overall structure of the loop is similar. For clarity, IgG2-IgG4 have not been shown, but their structures are similar to that for IgG1. (F) C_H_1 FG loop structure in IgG1 (PDB entry 6WFY, blue) and IgD (Fab^3^, grey). The overall structure of the loop is similar. For clarity, IgG2-IgG4 have not been shown, but their structures are similar to that for IgG1.

The C_H_1 BC loop comprises residues 153-158 (Figs. 3 and 4; residue numbering is according to the IgD Fab structures). In all structures that were compared from each set (IgA1, IgD, IgG1-4 and IgM), a proline residue is found at position 156, a *cis* peptide bond is found between residue 155 and Pro156 and the loop conformation for these residues is similar (Figs. 5A and 5B). The Cα1 (IgA1) and Cγ1 (IgG1-4) BC loops contain a second proline residue at position 158. The Cα1 domain contains a single-residue insertion (glutamic acid) between residues 157 and 158 and, even though the overall position of residue 158 is altered due to this insertion, a *cis* peptide bond is found between residue 157 and Pro158 in both Cα1 and Cγ1 domains (Figs. 5A-C). On the other hand, the Cμ1 BC loop contains a serine residue at position 158 and here the loop adopts a different conformation to that found in the Cα1 and Cγ1 domains, with a *trans* peptide bond between Asp157 and Ser158 (Fig. 5A). Like Cμ1, a serine residue is found at position 158 in the Cδ1 domain. However, in contrast to the Cμ1 BC loop, the Cδ1 BC loop contains a *cis* peptide bond between Thr157 and Ser158, and the loop adopts the same conformation as in the Cγ1 domain in all IgG subclasses (Figs. 5B and 5C). That IgD is more IgG-like could suggest that a second *cis* peptide bond is important to maintain the structure of the C_H_1 BC loop, which appears to be more conformationally restricted when another proline residue is present.

The C_H_1 DE loop comprises residues 177-186 (Figs. 3 and 4). The sequence for this loop differs substantially between the different isotypes and, compared with the Cδ1 and Cγ1 (IgG1-4) DE loops, the C∊1 and Cα1 (IgA1 and IgA2) loops contain one insertion, while the Cε1 loop has one deletion. However, a conserved tyrosine residue, which packs against the BC loop, is found in all isotypes at position 185 (Figs. 4 and 5D). Given the sequence diversity of the DE loop, it is perhaps unsurprising that it adopts a variety of different conformations in different isotypes. Intriguingly, however, the Cδ1 DE loop adopts a similar conformation to the Cγ1 DE loop in all IgG subclasses (Fig. 5E).

The C_H_1 FG loop comprises residues 209-212 (Figs. 3 and 4). Like the DE loop, the FG loop contains a conserved residue in all isotypes, a histidine at position 209 and, like the conserved tyrosine residue in the DE loop, His209 also packs against the BC loop (Fig. 5D). Within the short FG loop, IgG and IgM contain proline residues at position 211 and 210, respectively. In IgA1, a proline is found adjacent to the loop, at position 213. By contrast, a serine residue is found at this position in IgA2. A proline residue is likewise absent in IgD. Like the DE loop, the conformation of the FG loop shows structural diversity in different isotypes. However, here again the Cδ1 FG loop adopts a similar conformation to the Cγ1 FG loop in all IgG subclasses (Fig. 5F).

Packing interactions with the conserved residues Tyr185 and His209, and the presence of a proline residue at position 156, could restrict the conformation of the C_H_1 BC loop. The BC loop is close to the linker between the V_H_ and C_H_1 domains, with the implication that substantial conformational diversity within the BC loop could reduce the range of Fab elbow angles available to the V_H_-V_L_ and Cδ1-C_L_ domain pairs.

### 3.5 In IgD, the junction between the C_H_1 domain and upper hinge adopts a unique structure

**Fig. 6:**
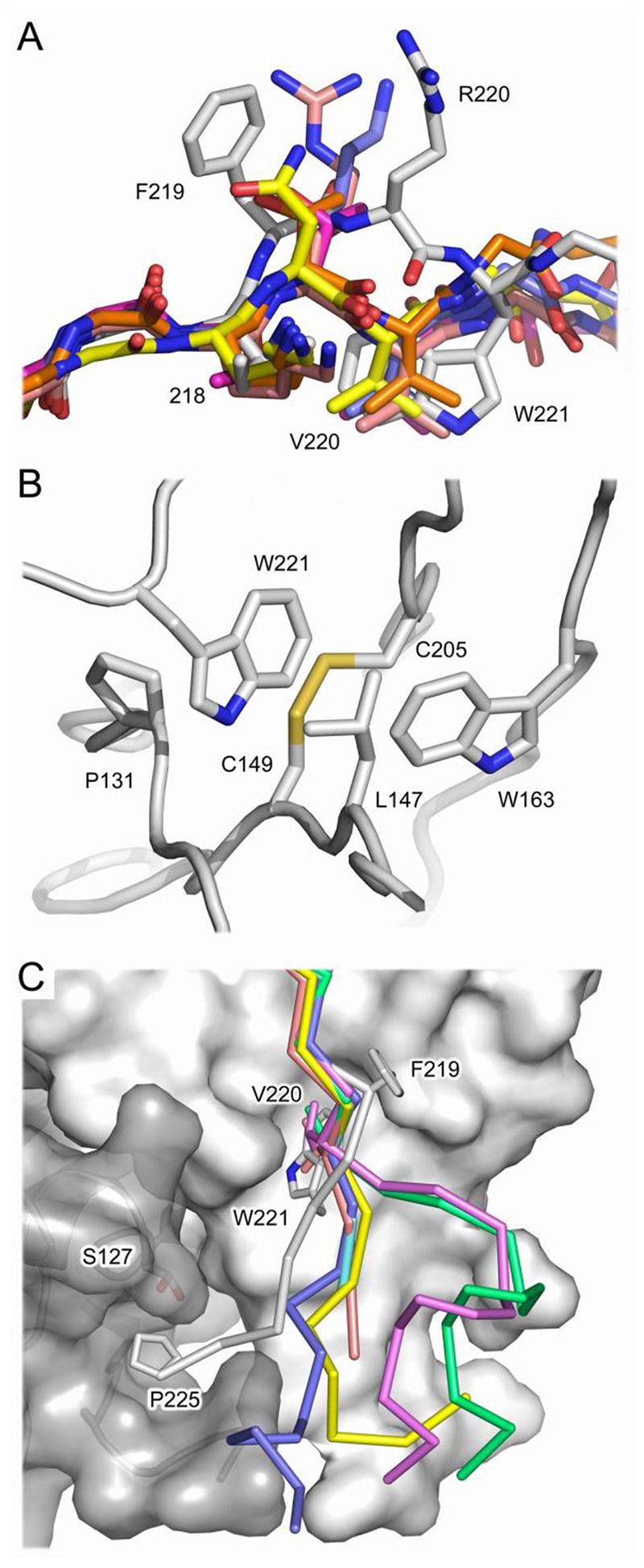
The junction between the C_H_1 domain and upper hinge adopts a unique structure in IgD. (A) The junction between the C_H_1 domain and upper hinge adopts a conserved structure in IgA1 (PDB entry 3M8O, purple), IgG1 (PDB entry 6WFY, blue), IgG2 (PDB entry 6TKD, orange), IgG3 (PDB entry 4M1D, salmon) and IgG4 (PDB entry 7VUX, green), which is similar to the IgM Fab (PDB entry 1DN0, yellow). In IgD (Fab^3^, grey), a conformational difference at Phe219 introduces a kink in the β-strand and Trp221 occupies a equivalent position to Val220 found in the other isotypes. For clarity, IgG2-IgG4 have not been shown, but their structures are similar to that for IgG1. (B) Trp221 environment in IgD. Trp221 is sandwiched between Pro131 and Leu147 and contacts Trp163, Cys149 and Cys205. (C) In intact IgG1 (PDB entry 1HZH, blue and yellow) and IgG4 (PDB entry 5DK3, green and purple; 6GFE, cyan and salmon) structures, the position of Val220 is conserved and the conformation of the hinge differs substantially only after this residue. In IgD (Fab^3^, grey), the junction between the Cδ1 domain and upper hinge adopts a different structure. In addition to the packing interactions formed by Trp221, Pro225 from the IgD upper hinge contacts Ser127 from the light chain. For IgD, the Fab^3^ structure is shown and the surface representations for the heavy and light chains are colored light grey and dark grey, respectively. For all structures, β-strand G Cα atoms are shown. The Cδ1 and Vλ domains have been shown as a surface representation. For clarity, part of the Cδ1 AB and EF loops have not been shown.

In IgA1, IgD and IgG1-4, the junction between the C_H_1 domain and upper hinge is located within β-strand G. In IgM, the sequence of the Fab extends just beyond this strand (Fig. 4). When a set of crystal structures was compared for each isotype, it was found that the structure of the C-terminal region of the C_H_1 domain and junction with the hinge was similar within the set. Furthermore, the structure of this region was conserved between IgA1 and IgG1-4, and was similar in IgM (Fig. 6A). By contrast, the structure of the C-terminal region of the C_H_1 domain and junction with the hinge differed substantially in IgD (Fig. 6A).

In all five independent views of the IgD Fab the direction of the main chain is altered for IgD, compared with the other isotypes, at residue Phe219 at the C-terminus of the Cδ1 domain, and a kink is introduced into β-strand G (Fig. 6A). As a result, Arg220, the first residue of the IgD upper hinge, faces away from the Cδ1 domain instead of packing against the C_H_1 domain like the structurally conserved residue Val220 found in IgA, IgG and IgM. Instead, in IgD, Trp221 from the upper hinge occupies a structurally equivalent position to Val220 and forms more substantial interactions with conserved C_H_1 domain residues: Trp221 is sandwiched between Pro131 and Leu147 (Ile147 in IgA1 and Val147 in IgM), and contacts Trp163, in addition to Cys149 and Cys205 that form a disulfide bond (Figs. 6A and 6B). Furthermore, Pro225 from the upper hinge packs against Ser127 from the λ light chain (Fig. 6C). A similar interaction could also form between Pro225 and a κ light chain, in which Ser127 is replaced by aspartic acid.

Trp221 is close to a region of the Cδ1 domain that is observed in multiple conformations. In Fab^1B^ and Fab^3^, the Arg137 side chain forms a hydrogen bond with the Trp221 main chain; this interaction is absent in Fab^1A^, Fab^2^ and Fab^4^ as Arg137 either adopts a different conformation or is partially disordered. Thus, the conformation adopted by Trp221 appears to be independent of conformational changes in the Cδ1 AB loop (Figs. 2B and 2C). Furthermore, the kink in β-strand G at the C-terminus of the Cδ1 domain (Phe219) appears to be unique to IgD; it was not observed in the set of structures of the other human isotypes.

In intact IgG1 and IgG4 structures (Blech et al., 2019; Saphire et al., 2001; Scapin et al., 2015), the Val220 main chain at the C-terminus of the Cγ1 domain adopts a conserved conformation, and the structure of the upper hinge differs substantially after this residue (Fig. 6C). By contrast, the conformational change about Phe219, and the resulting packing interactions formed between Trp221 from the upper hinge and the Cδ1 domain, and Pro225 and the λ light chain, could restrict the flexibility and conformation of the upper hinge in IgD.

Of the human antibody isotypes, IgG3 and IgD have the longest hinge regions with 62 and 64 residues, respectively. The IgG3 hinge contains eleven cysteine residues that form inter-chain disulfide bonds. Solution-based biophysical techniques have shown that IgG3, and by implication the IgG3 hinge, adopts an extended, elongated structure in solution (Spiteri et al., 2021). By contrast, the IgD hinge contains a single cysteine residue and solution-based techniques have shown that IgD is predominantly T-shaped, yet flexible, with a semi-extended hinge (Sun et al., 2005). Although the hinge regions of these two isotypes are markedly different, the unique conformation of the upper hinge in IgD, and the packing interactions with the Cδ1 domain and λ light chain, could affect the overall disposition of the Fab fragments with respect to the Fc region: a conformationally restricted upper hinge could contribute to the predominant T-shape of human IgD.

Trp221 and Pro225 from the upper hinge are conserved in primate species including chimpanzee, baboon, mangabey, rhesus macaque, cynomolgous macaque (Rogers et al., 2006) as well as orangutan and gibbon (Fig. S1). It remains to be determined whether IgD antibodies in which Trp221 and Pro225 are conserved adopt the predominantly T-shaped structure observed for human IgD.

## 4 Conclusion

In summary, we solved crystal structures of the IgD Fab, providing five independent views of the structure of the Cδ1 domain. These structures reveal conformational diversity in specific regions of the Cδ1 domain, particularly in the AB and EF loops. Comparisons with C_H_1 domain structures of other isotypes provide support for the idea of an evolutionary link between IgD and IgG. Among the human isotypes, the structures also reveal features unique to IgD, such as the upper hinge region, that could play a role in determining its overall structure.

## Supplementary Material

Fig. S1

## Figures and Tables

**Table 1:** Data processing and refinement statistics.

Data Processing				
	Fab^1^	Fab^2^	Fab^3^	Fab^4^
Space group	*P* 1	*P* 2_1_	*P* 2_1_	*P* 2_1_ 2_1_ 2_1_
*a, b, c* (Å)	43.53, 71.79, 90.86	43.15, 73.83, 70.46	42.89, 74.20, 70.91	57.77, 71.64, 131.63
*α, β, γ* (°)	92.52, 91.16, 105.33	90.00, 92.54, 90.00	90.00, 92.15, 90.00	90.00, 90.00, 90.00
Resolution (Å)^a^	69.15-2.75 (2.88-2.75)	70.39-1.55 (1.58-1.55)	70.87-1.45 (1.47-1.45)	71.64-2.10 (2.16-2.10)
Completeness (%)^a^	98.9 (98.5)	100.0 (100.0)	100.0 (100.0)	99.9 (99.2)
Multiplicity^a^	3.5 (3.2)	7.0 (6.2)	6.5 (5.1)	7.3 (7.4)
Mean (I)/σ(I)^a^	8.1 (1.1)	10.0 (1.3)	11.2 (1.2)	9.4 (1.5)
CC_1/2_^a^	0.997 (0.776)	0.998 (0.486)	0.999 (0.462)	0.998 (0.627)
*R*_pim_(%)^a^	4.6 (50.4)	4.0 (92.3)	3.2 (66.1)	5.0 (57.0)
*R*merge(%)^a^	7.2 (76.2)	9.9 (212.5)	7.7 (134.5)	12.6 (147.0)
Wilson *B* factor (Å^2^)	72.92	18.02	15.11	37.27
**Refinement**				
R_work_ / R_free_(%)^b^	21.61 / 25.58	16.06 / 19.76	15.68 / 18.93	17.89 / 21.63
No. of reflections	27 159	63 950	78 598	32 645
RMSD				
Bond lengths (Å)	0.003	0.007	0.007	0.003
Bond angles (°)	0.554	0.941	0.932	0.705
Coordinate error (Å)	0.48	0.18	0.16	0.26
No. of atoms				
Protein	6583^c^	3 465^c^	3 484^c^	3385^c^
Solvent	13	337^d^	387^d^	197
Other	9	51^e^	48^f^	67^cg^
Average B factor (Å^2^)				
Protein	100.52	25.66	25.57	44.91
Solvent	79.95	36.37	36.85	46.13
Other	118.32	53.51	48.39	58.81
Ramachandran plot				
Favored (%)	95.82	98.17	98.84	95.86
Allowed (%)	4.18	1.83	1.16	3.68

a Values in parentheses are for the outer shell

b R_free_ set comprises 5% of reflections

c Includes alternative conformations

d Includes alternative positions

e Azide, ethylene glycol, nitrate, sodium and tris

f Ethylene glycol, polyethylene glycol and sodium

g Chloride, ethylene glycol and polyethylene glycol

## Data Availability

Coordinates and structure factors have been deposited at the Protein Data Bank with accession numbers 8OJS (Fab^1^), 8OJT (Fab^2^), 8OJU (Fab^3^) and 8OJV (Fab^4^).
