# Supplementary material for: Crystal structures of the human IgD Fab reveal insights into CH1 domain diversity
Source: Mol Immunol. Author manuscript; Available in PMC 2023 Jul 1. (PMC7614686; doi:10.1016/j.molimm.2023.05.006)
Supplement: Fig. S1 [file EMS176659-supplement-Fig__S1.pdf]

Supplemental Information

Crystal structures of the human IgD Fab reveal insights into C_H1 domain diversity

Anna M. Davies[a], Rebecca L. Beavil[ab], Momchil Barbolov[ac], Balraj S. Sandhar[ad], Hannah J. Gould[a], Andrew J. Beavil[a], Brian J. Sutton[a] and James M. McDonnell[a]

Author Affiliation:

[a] King's College London, Randall Centre for Cell and Molecular Biophysics, New Hunt's House, London, SE1 1UL, United Kingdom.
[b] Current address: 272BIO Limited, The Pirbright Institute, B-Block, Ash Road, Pirbright, Woking, Surrey, GU24 0NF, United Kingdom.
[c] Current address: Medical University of Varna, Faculty of Pharmacy, Department of Biochemistry, bul. Tsar Osvoboditel 150, Varna, 9002, Bulgaria.
[d] William Harvey Research Institute, Barts and The London School of Medicine and Dentistry, Queen Mary University of London, London, EC1M 6BQ, United Kingdom.

Corresponding author:

James M. McDonnell
King's College London
Randall Centre for Cell and Molecular Biophysics
New Hunt's House
Guy's Campus
London
SE1 1UL
United Kingdom

Tel: + 44 (0) 20 7848 6970
E-mail: james.mcdonnell@kcl.ac.uk

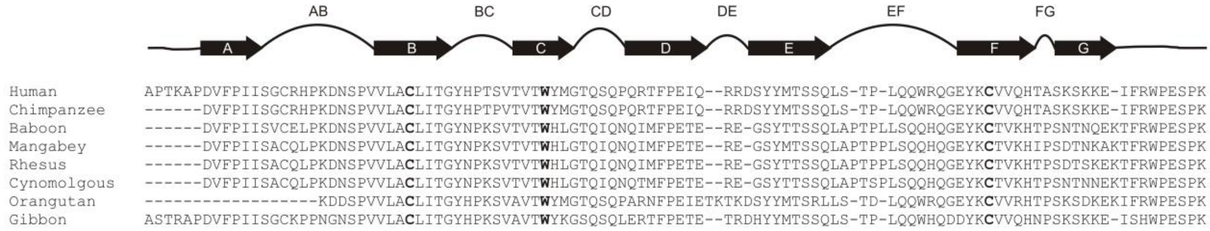

Fig. S1. Alignment of non-human primate IgD C_H1 domain sequences. Sequences were obtained from GenBank (Sayers et al., 2020) and UniProt (The UniProt Consortium, 2021) with the following accession numbers: human, UniProt P01880; chimpanzee, GenBank DQ297174; baboon, GenBank DQ297176; mangabey, GenBank DQ297182; rhesus macaque, GenBank DQ297179; cynomolgous macaque, GenBank DQ297178; orangutan, UniProt A0A8I5TYP2; gibbon, UniProt A0A2I3H7W1. The sequence alignment was performed using Clustal Omega (Madeira et al., 2022). Approximate positions of β-strands are indicated, based on the human structure. Highly conserved amino acids of the 'central pin' residues of Ig-domains (Williams and Barclay, 1988) are in bold and coloured black.

Supplemental references:

Madeira, F., Pearce, M., Tivey, A.R.N., Basutkar, P., Lee, J., Edbali, O., Madhusoodanan, N., Kolesnikov, A., Lopez, R., 2022. Search and sequence analysis tools services from EMBL-EBI in 2022. Nucleic Acids Res. 50, W276–W279.

Sayers, E.W., Cavanaugh, M., Clark, K., Ostell, J., Pruitt, K.D., Karsch-Mizrachi, I., 2020. GenBank. Nucleic Acids Res. 48, D84–D86.

The UniProt Consortium., 2021. UniProt: the universal protein knowledgebase in 2021. Nucl. Acids Res. 49, D480-D489.

Williams, A.F., and Barclay, A.N., 1988. The immunoglobulin superfamily - domains for cell surface recognition. Annu. Rev. Immunol. 6, 381-405.
